# A Visual Sensing Concept for Robustly Classifying House Types through a Convolutional Neural Network Architecture Involving a Multi-Channel Features Extraction

**DOI:** 10.3390/s20195672

**Published:** 2020-10-05

**Authors:** Vahid Tavakkoli, Kabeh Mohsenzadegan, Kyandoghere Kyamakya

**Affiliations:** Institute for Smart Systems Technologies, University Klagenfurt, A9020 Klagenfurt, Austria; kabehmo@edu.aau.at (K.M.); kyandoghere.kyamakya@aau.at (K.K.)

**Keywords:** classification, house architecture type classification, house type classification, convolutional neural networks

## Abstract

The core objective of this paper is to develop and validate a comprehensive visual sensing concept for robustly classifying house types. Previous studies regarding this type of classification show that this type of classification is not simple (i.e., tough) and most classifier models from the related literature have shown a relatively low performance. For finding a suitable model, several similar classification models based on convolutional neural network have been explored. We have found out that adding/involving/extracting better and more complex features result in a significant accuracy related performance improvement. Therefore, a new model taking this finding into consideration has been developed, tested and validated. The model developed is benchmarked with selected state-of-art classification models of relevance for the “house classification” endeavor. The test results obtained in this comprehensive benchmarking clearly demonstrate and validate the effectiveness and the superiority of our here developed deep-learning model. Overall, one notices that our model reaches classification performance figures (accuracy, precision, etc.) which are at least 8% higher (which is extremely significant in the ranges above 90%) than those reached by the previous state-of-the-art methods involved in the conducted comprehensive benchmarking.

## 1. Introduction

**Figure 1:**
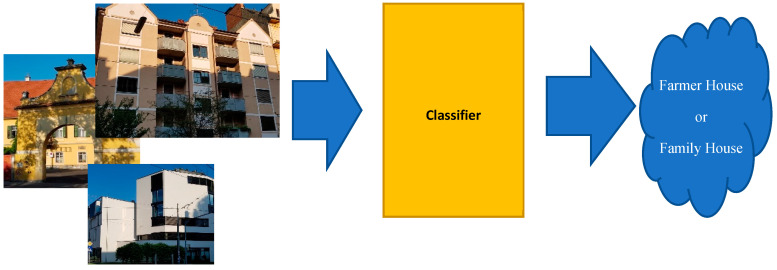
The “House type” classification’s overall process pipe (Source: own images).

Most visual sensors integrate an image classification related functional bricks. Indeed, image classification is one of the branches of computer vision. Images are classified based on the information abstracted from a series of sequential functional processes, which are preprocessing, segmentation, feature extraction, and finding best matches [1]. Figure 1 roughly illustrates both the input (s) (i.e., an image or some images) and the output of the classifier module. It gets a color image as input and it returns the house-type label, which may be, for example, a bungalow, a villa, a one-family house, etc. Various factors or artefacts in the input images may result in a significant reduction of the classification confidence. Some examples: artifact in image like garden, poor view of image or their neighbor’s houses. Worth mentioning is that object classification from visual sensors generated images is a functional brick of high significance in a series of very practical and useful use cases. Some examples of use-cases, just to name a few, are found in real-world robotic applications, such as image/object recognition [2], emotion sensing [3], search and rescue missions, surveillance, remote sensing, and traffic control [4].

Automatically recognizing the architectural type of a building/house from a photo/image of that building has many applications such as an understanding of the historic period, the cultural influence, a market analysis, city planning, and even a support of the price/value estimation of a given building [5,6,7].

Various candidate known image classification concepts/models can be used for performing this house classification endeavor.

Thus, as we have a classifier model, the model should be optimized w.r.t. to a related loss function. In this case, we use one of the most famous loss functions, which has been often used for classifications tasks, the so-called categorial cross entropy [8,9]. Equation (1) presents this chosen loss function:

(1) $$L\left(y,\hat{y}\right)=\;-\;\frac{1}{N}\overset{M}{\underset{j=1}{\sum }}\overset{N}{\underset{i=1}{\sum }}\left[y_{i,j}\log\left({\hat{y}}_{i,j}\right)\right]$$

where L is the chosen loss function; *N* is the number of class categories; *M* is the number of samples; $y_{i,j}$ relates to the different true labels; and ${\hat{y}}_{i,j}$ relates to the different predicted labels. During the training process, the model will be optimized in a way such that the minimum value of the objective function L in Equation (1) is reached. Subsequently, the model shall be tested and verified. 

There are several traditional image classification schemes such as SVM (support vectors machine), just to name one, which can theoretically/potentially be used [10]. However, most of them are not robust enough to capture and learn the relatively very complex patterns involved here in the house classification task although some of them (e.g., SVM) have been proven to be universal approximators. Therefore, one should use/involve truly much high-performing concepts to solve this very difficult/challenging classification task at hand [10]. It is also shown that combining those traditional methods with dynamical neural networks like cellular neural networks can result in a significant performance improvement. For example, Al Machot et al. [11] showed that combining SVM with cellular neural networks considerably improves the SVM performance; this new resulting hybrid model can thus be used as a very fast and robust detector/classifier instead of using the sole SVM model. 

In the recent years, the use of convolutional neural networks (CNN) has been increasing at a fast rate for classification and various data processing/mining tasks [12,13,14,15,16,17,18,19]. The input/output data can be represented as arrays or as multi-dimensional data like images. At the heart of a CNN network, we have convolution operators by which the input values in each layer are convoluted with weights matrices [20]. After/before these operations, other operations like sub-sampling (e.g., Max-pooling) or “batch normalization” can be used [17,21]. This process can be repeated and thereby creates several layers of a deep neural network architecture. The last layer is finally connected to a so-called “fully connected” layer. In addition, the network can have some additional channels for different features like putting RGB channels or an edge or blurred image as additional channels [22,23,24,25,26]. The main idea behind this complex structure is based on filtering non-appropriate data. Each filter which is applied will remove some uninteresting/non-appropriate data. Therefore, it results into a smaller network structure and thus the training requires less time as this technique will shrink the searching area. 

The Convolutional Neural Network concept was first introduced by Yann LeCun et al. [17] in the 1980s. This model has been created based on both convolutional and sub-sampling layers. Although this model was introduced in the 1980s, it was not yet used popularly in the first years, as computing’ processing power and other resources were still very restricted and limited. But nowadays, those restrictions have been removed due to the recent “computing”-related technological advances/progress and one has seen various usages/applications of such neural networks for significantly large problems.

The model developed and used in this paper is based on a CNN architecture, whereby, however, features are extracted through different input channels. In Section 2, we briefly discuss some related works of relevance for house classification. Our novel model is then comprehensively explained in Section 3. Thus, in Section 4, our model is tested and compared with another relevant models while using/involving the very same test data and the results obtained are comprehensively analyzed and discussed. To finish, in Section 5 concluding remarks are summarized.

## 2. Related Works

**Figure 2:**
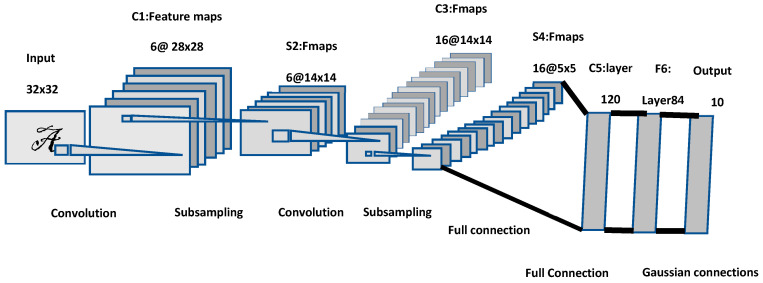
Architecture (our own redrawing) of the LeNet-5 model [27].

Numerous approaches for image classification have been presented over the years. In 1998, LeCun et al. [27] presented a convolutional neural network model to classify handwritten digits. This model (called LeNet-5) comprises three convolutional layers (C1, C3, C5), two average pooling layers (S2, S4), one fully connected layer, and one output layer (see Figure 2). This model involves sigmoid functions to include/consider nonlinearity before a pooling operation. The last layer (see output layers) is using a series of Euclidian Radial Basis Function units (RBF) [28] to classify 10 digits amongst 10 possible classes.

LeNet-5 and LeNet-5-(with distortion) reached after extensive experiments an accuracy of 0.95% and 0.8%, respectively, on the MNIST data set. However, by increasing both the resolution of an image and the number of classes of a classification endeavor, the machine needed for computing consequently requires more powerful processor systems (e.g., GPU units) and a much deeper convolutional neural network model.

In 2006, Geoffery Hinton and Salakhutdinov showed that the neural network with multiple hidden layers can improve the accuracy of classification and prediction by improving different degrees of abstract representation of the original data [29].

**Figure 3:**
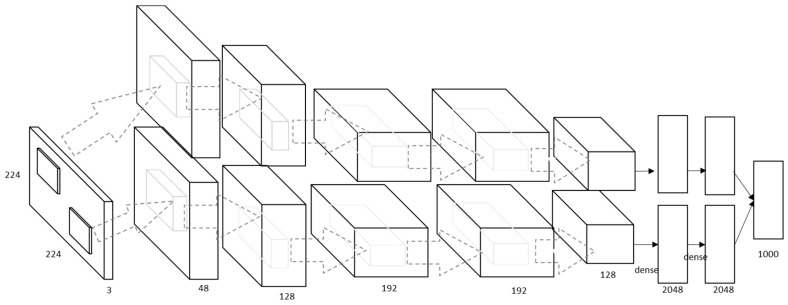
Architecture (our own redrawing) of AlexNet [30].

In 2012, Krizhevky et al. [30] introduced a large deep CNN (AlexNet). The AlexNet model is much bigger than LeNet-5 with the same acritude (see Figure 3). This model has 5 convolutional layers and 3 fully connected (*FC*) layers. The rectified linear unit (*ReLU*) and the FC layers enables the model to be trained faster than similar networks with *tanh* activation function units. They also added a local response normalization (*LRN*) after the first and the second convolutional layer; that enables the model to normalize information. They further added a max-pooling layer after the fifth convolutional layer and after each *LRN* layer. The stochastic gradient descent (SGD) method has been used for training the AlexNet with a batch size of 128, a weight decay of 0.0005 and a momentum of 0.9. The weight decay works as a regulator to reduce the training error.

Also, Jayant et al. [31] presented a model to capture the structural relationships based on statistics of raw-image-patches in different partitions of a document-image. They compared the Relevance Feedback (RF) model to the Support Vector machine (SVM) model and reported that whenever the number of features is large, a combination of SVM and RF is more suitable.

**Figure 4:**
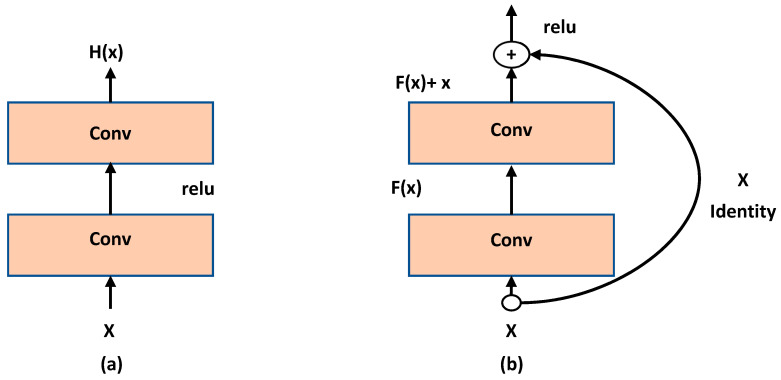
The ResNet model (our own redrawing)—(**a**) Plain layer; (**b**) Residual block [32].

In 2016, He et al. [32] proved that increasing the depth of a CNN processor with more layers increases model complexity on one hand and decrease convergence rate on the other hand. The main problem happens due to introducing new intermediate weights and a consecutive training need to optimize them. For solving this problem, they suggested creating a shallower model with additional layers to perform an identity mapping. Figure 4 shows their core approach. 

The H(x) block is defined as H(x)=F(x)+x. Therefore, F(x)+x will be encapsulated as one block H(x) and the internal complexity of this block shall be hidden. This model is called ResNet and it did show 6% to 9% of accuracy error in classification against the CIFAR-10 test set.

**Figure 5:**
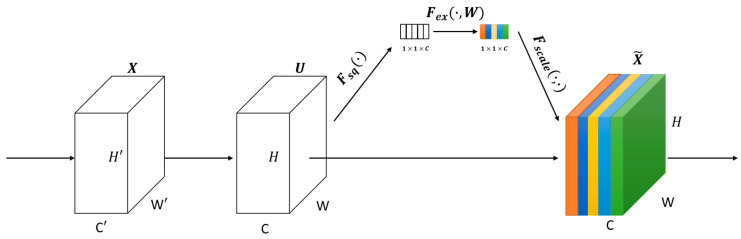
SENet (our own redrawing)—A Squeeze and excitation block [33].

Later, the encapsulation layers concept was extended [33] by introducing a so-called Squeeze-and-Excitation network (SENet). This model reduces the top-5 classification error to 2.25%. The main architecture of this model is shown in Figure 5. Each block is composed of four functions. The first function is a convolution ($F_{tr}$). The second function is a squeeze function ($F_{sq}$) which performs an average pooling on each of the channels. The third function is an excitation function ($F_{ex}$) which is created based on two fully connected neural networks and one activation function (ReLu). The last function is a scale function to generate the final output ($F_{scale}$). It is known that SENet has shown/demonstrated very good performance results compared to previous models in terms training/testing time and accuracy.

Regarding house classification, a careful study of previous works shows that an automatic detection of architectural styles, and furthers, much harder, even of house types/classes is not yet very well developed/researched [34]. Only few studies on the matter have been published so far. Mathias et al. [35] published a work using SVM to distinguish 4 classes of architectural style, with a specific focus on “inverse procedural modeling”—thereby using imagery to create a generative procedural model for 3D graphics. 

**Figure 6:**
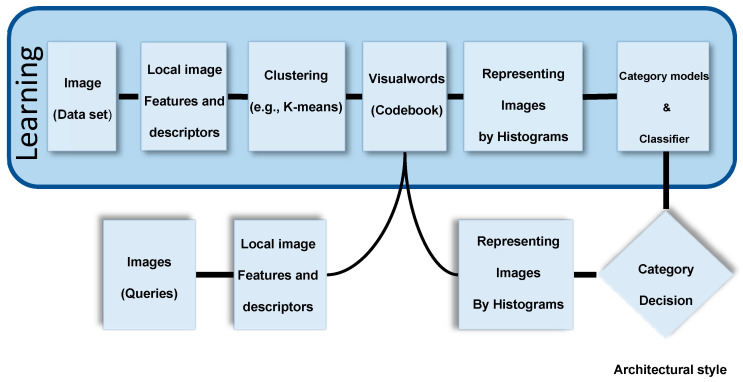
Learning visual words and classification (our own redrawing) scheme [36].

Shalunts et al. [36] published a further work to classify the architectural styles of facade windows (see Figure 6). They did thereby use a relatively small dataset (i.e., 400 images) for classifying the architectural styles of buildings through related typical windows in three classes which are: Romanesque, Gothics, and Baroque. Ninety images of the dataset were used for training (i.e., 1/3 of the data of each class).

**Figure 7:**
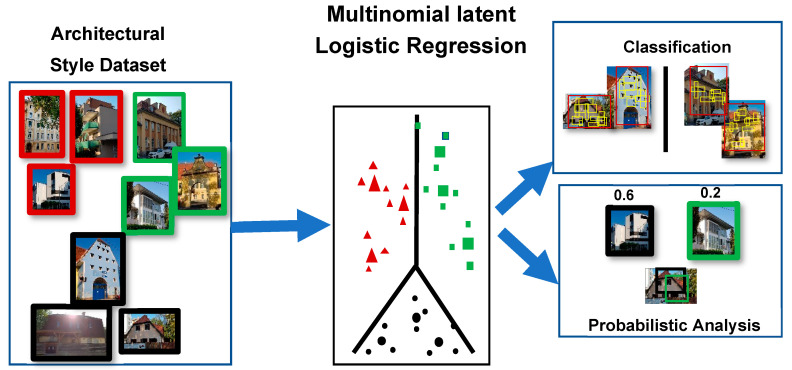
Schematic illustration (our own redrawing) of an architectural style classification using the Multinomial Latent Logistic Regression (MLLR) [37].

Xu et al. [37] developed a 25-class dataset from Wikimedia and used a model involving HOG that classified through the Multinomial Latent Logistic Regression (see Figure 7). Their model was able to find the presence of multiple styles in the same building through a single image. Notably, they included the “American Craftsman” (one of the house styles used in this work) as a class. Both groups (of authors) lastly mentioned noted the acute absence of a publicly available dataset for architectural style recognition.

**Figure 8:**
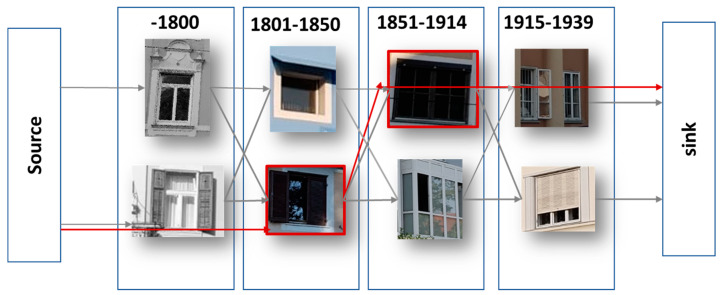
Sample chain graph (our own redrawing). Elements in adjacent periods are fully connected with weights depending on their co-occurrence, while the source and sink connect to every node with weights that penalize the number of skipped periods. Here, the shortest path (in red) skips pre-1800 and 1915–1939 because they lack the long balconies of the other periods. (For clarity, this visualization shows only four periods (instead of ten), and only some source and sink edges [38]).

In 2015, Lee et al. [38] published a work in which they have used a large dataset of nearly 150 k Google Street View images of Paris, combined with a real estate cadaster map to date building façades and discover the evolution of architectural elements over time (see Figure 8). Their approach used HOG descriptors of image patches to find features correlated with a building’s construction time period. 

**Figure 9:**
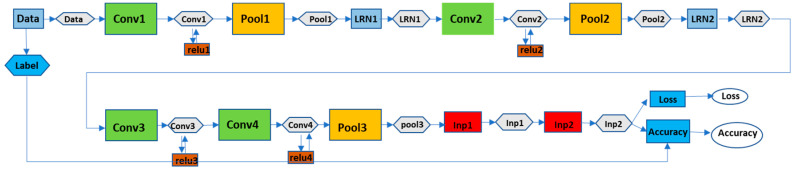
CNN’s architecture (our own redrawing), conformed by four convolutional layers, three pooling layers, two normalization layers and two fully-connected layers at the end [39].

In 2016, Obeso et al. [39] presented a work based on convolutional neural network (CNN) using sparse features (SF) to classify images of buildings in conjunction with primary color pixel values (see Figure 9). As a result, their mode achieved of 88.01% accuracy.

We conclude from previous studies that house classification requires very sophisticated classifier models, which shall cover all aspects of the related problem/task and it further becomes evident that CNN is very good candidate for filling this gap (i.e., solving this tough classification task).

## 3. Our Novel Method/Model

**Figure 10:**
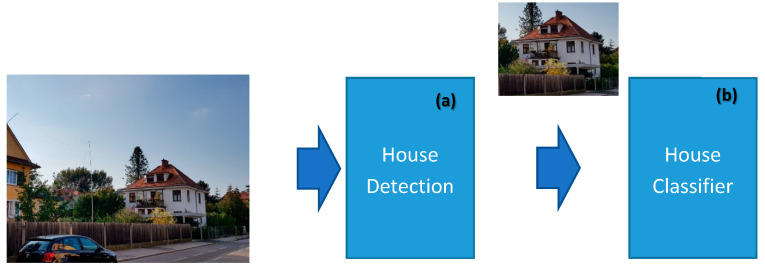
The novel global model is composed of (**a**) house detection and (**b**) classification modules. (Source: our own images).

The basic problem formulation has been graphically presented in Figure 10 which essentially underscores the goal of the CNN deep neural model to be developed. However, for reaching the goal with a sufficient accuracy, a series of problems related to the quality of the input “house images” must be solved. 

**Figure 11:**
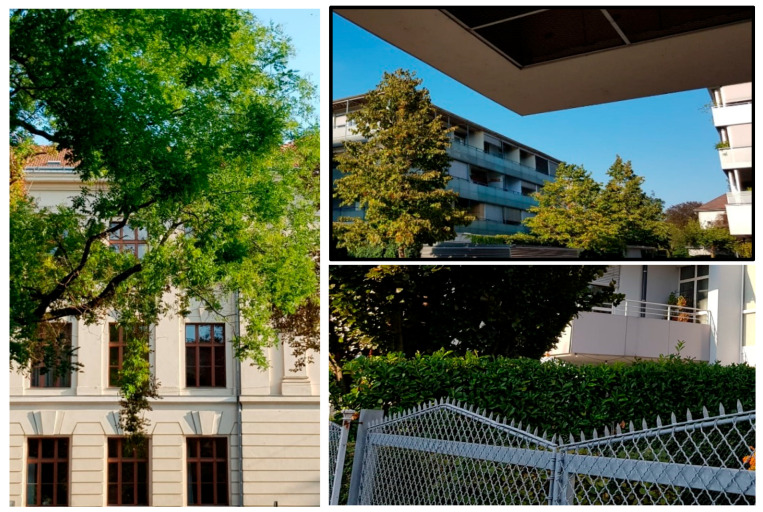
Image problems’ illustration: poor view/perspective, more pool garden and/or pool instead of a view of the house, etc. (Source: our own pictures).

These problems/issues can be grouped into three different categories (see Figure 11):

- Images, which do not contain a house but only some additional information like garden or trees, make the house classification difficult. Such images are not appropriate for use for a house classification endeavor.
- Some images are (maybe) captured from a very poor angle of the house and thus the house is not well recognizable on them.
- Some house classes have strong similarities with other classes; this is a potential source of misclassification amongst them.

For solving the mentioned problems, our overall model (see Figure 11) is designed with two modules: (a) a house detection module, and (b) a house classifier module.

The house detection module is responsible for finding/detecting/localizing a house and its bounding box within the input image. Thus, the result of this module is a bounding box in the input image. It shall also inform us on how much the image has a similarity to a house if at all. This module/layer helps the classifier to perform much better. The second module/layer is for house classification. It may consider all the image or, depending on the outcome of the first module, consider only an image portion within the bounding box identified by the first module/layer. In the lastly mentioned case, the image portion is cropped from the original input image and it becomes the input to be given to the second module for classification. 

### 3.1. House Detection

**Figure 12:**
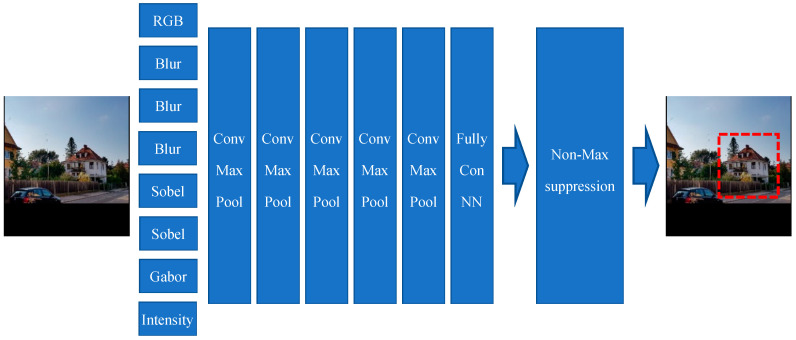
House detection model based on a convolutional neural network. The output of the convolutional neural network will be 4 boundary boxes with four house similarity factors. The boundary boxes with house similarity of 95% will be selected for Non-Maximum suppression with 0.65 overlapping threshold. The house boundary box will be the output of the Non-Maximum suppression module. (source of input image: our own image).

**Figure 13:**
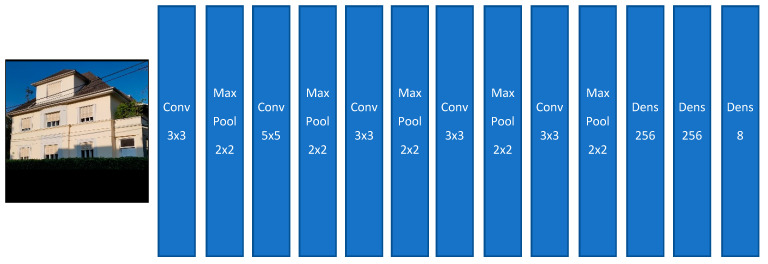
House classification Model I (Source of input image: our own image).

**Figure 14:**
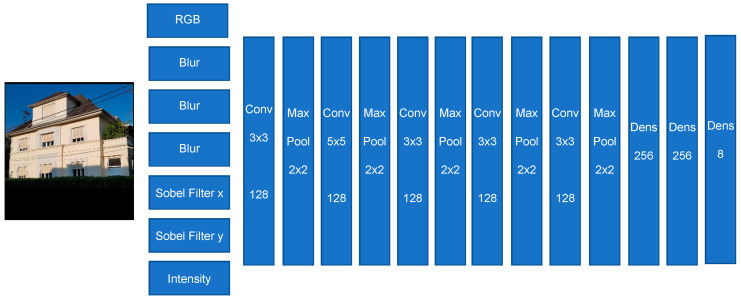
The house classification Model II (Source of input image: our own image).

**Figure 15:**
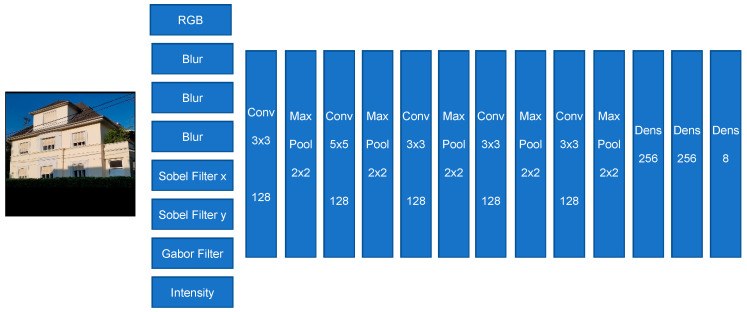
Modell III—Convolutional neural network for house classification. The output of the model consists of 8 house classes (Source of input image: our own image).

As explained previously, some images contain either very poor views of the house or/and some additional, for the classifier non-relevant information. Those issues result in decreasing both precision and accuracy of our classifier module. Therefore, this module is responsible for finding the image portion(s) which is/are house views and crop it/them. Figure 11 shows the overall house detection model. The input image is of size 200 × 200 with three channels. As input images may have different sizes, each original input image must therefore first be rescaled such as to fit either the width or the heights of 200 pixel; the rest of the image may have no values if the image is not a square ( i.e., or rectangular form). Therefore, the other parts (with no values) will be black in that case (rectangular form of an original input image). The output of this model (see Figure 12) is one boundary or bounding box. The image portion surrounded by the detected “boundary box” will be cropped out and it will the “input image” for the different classifier models described in Figure 13, Figure 14 and Figure 15 and the other models involved in the benchmarking process shown in Section 4. 

The house detection model contains three main parts: neural layers, feature extraction layers, and a Non-Maximum suppression layer. The feature extraction layers/channels (pre-processors) contain different well-known filters, such as the following ones: Blur filter, Sobel filter, and Gabor filter. These pre-processing filters help/support the model in taking more attention to aspects of the input image which are more important and much relevant. It is the convolutional neural network which is finding the house boundaries. The last part of the CNN architecture is responsible for creating the final boundary boxes by selecting a bounding box with 95% or a higher similarly factor and create the final boundary box based on the Non-Maximum Suppression Algorithm with 0.65 overlap threshold.

### 3.2. House Classification

The house classification module is designed to classify the input house images into eight different types. Figure 13 shows the overall house classification model. The input image is 200 × 200 with three channels. Cropped images from the previous module are first rescaled to fit either its width or its heights in 200-pixel square, and the rest of the model’s input square (of 200 × 200) has no values. Therefore, those rest parts of the input square are black. The output of this model is a class number/label. 

On the way to developing the very best model for house classification, we created several models from which to then select the best suitable one for the task at table. These different models are explained in this section.

#### 3.2.1. Model I

Our first classification model is composed of five convolutional layers. The outputs of those convolutional layers go into different max-pool layers. Finally, the output of the last max-pool layer goes into a dense layer, whereby the latest dense layer has eight output neurons, which are representing the eight house classes (see Figure 13).

#### 3.2.2. Model II

The second model, like the previous model, has five convolutional layers. The result/output of those convolutional layers will go respectively into max pool layers. Finally, the output of the latest max pool layer will go into the dense layers. The final dense layer has eight output neurons, which represent our eight house classes. The main difference between these two classifier models are the preparation/pre-processing layers of this second model.

These pre-processing layers of this second model provide/generate more details and they are indeed new channels besides the basic the color channels of the input image. These new additional channels are respectively: Blur 3 × 3, Blur 5 × 5, Blur 9 × 9, Sobel Filter X, Sobel Filter Y, and Intensity (see Figure 14).

#### 3.2.3. Model III

This model has also two main parts: a) neural layers, and b) features extraction layers. The features extraction pre-processing layers/channels contain different well-known filters such as the following ones: Blur, Sobel, and Gabor filters (see Figure 15). Here too, these pre-processing filters help/support the model in placing more attention on aspects of the input image, which are more important and relevant for the classification task.

**Figure 16:**
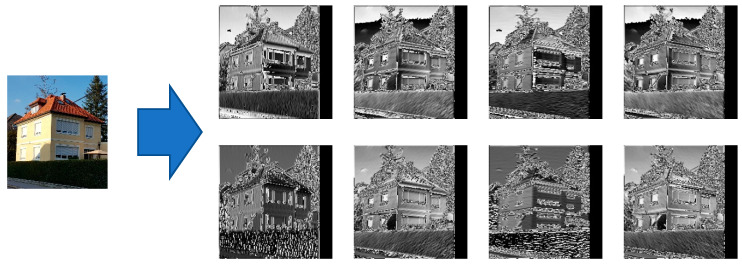
Effect of the Gabor filters on an input house image: The top row images are produced by Gabor filters with a kernel size 5, sigma 2, and theta having the following respective values: 0, 45, 90 and 135 degrees (from left to right). The bottom row images are produced by Gabor filters with kernel size 5, sigma 5, and theta having the following respective values: 0, 45, 90 and 135 degrees (from left to right). (Source of input image: own image).

Indeed, the pre-processing filters provide more relevant features to the model, and this significantly supports the training process to search and find those features, which are pointing directly to those parts of the input image, which are most relevant. Figure 16 shows, for illustration, the results of the image filtering through one of the pre-processing modules, here the Gabor filters. Each Gabor filtered image is highlighting some interesting features of the image which may help the classifier to better perform the classification task. 

## 4. Results Obtained and Discussion

**Figure 17:**
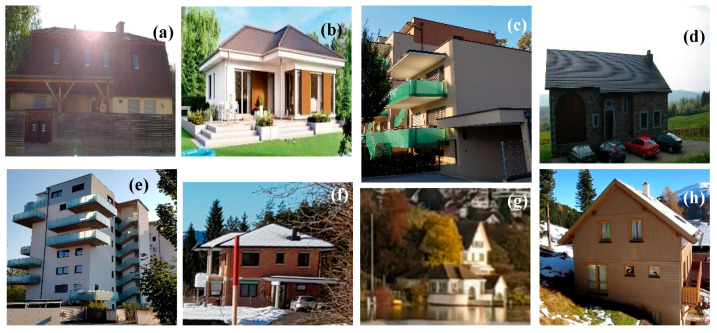
House types which are considered in this work—here some illustrative examples: (**a**) is Farmer house; (**b**) is bungalow; (**c**) is a duplex house; (**d**) is a detached house; (**e**) is an apartment house; (**f**) is a row house; (**g**) is a villa; (**h**) is a country house. (Source of input image: our own images).

As previously explained, several images were gathered from the Internet and used for both training and testing after an appropriate labelling: a total of 1200 images; the number of classes was 8 (see Figure 17 for illustration). 

The developed deep-learning model (made of two modules: see Figure 12 and Figure 15) was trained with 600 images and verified with 200 images and tested with 400 other images. Figure 14 shows the classification confusion matrix with 200 test images obtained by the best classification model (Figure 12 and Figure 15). 

All classifier models have been implemented on a PC with Windows 10 Pro, Intel Core i7 9700K as CPU, double Nvidia GeForce GTX 1080 TI with 8GB RAM as GPU and 64GB RAM. Here, the training takes approximately 8 h.

In order to understand and find an objective justification of why the best model is outperforming the other ones, we conduct a simple feature significance analysis. Hereby, we use the so-called NMI (normalized mutual information) for the input features. Table 1 shows the Normalized Mutual Information (NMI) scores obtained for the input features. It is clearly shown that by adding more specific features through the multi-channel pre-processing units/modules, the NMI is thereby respectively significantly increased. 

**Figure 18:**
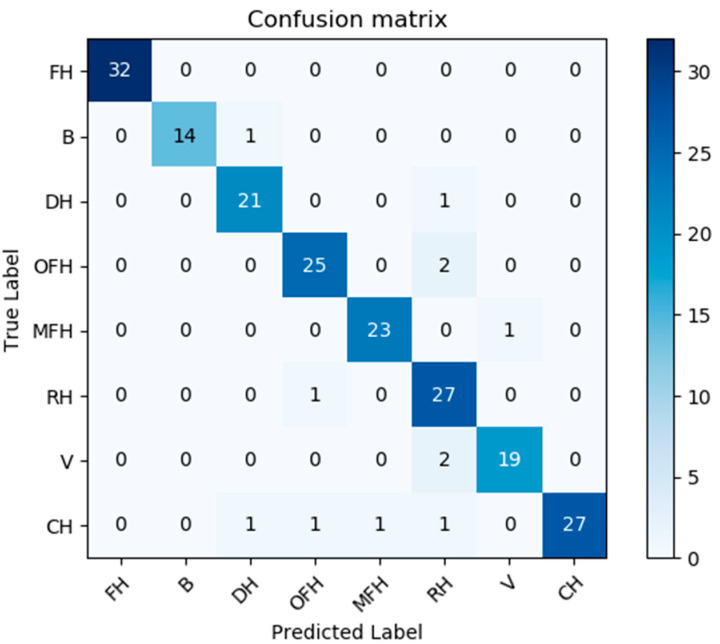
Confusion matrix of the results obtained from Model III while using 200 test images.List of classes: FH is farmer house; B is bungalow house; DH is duplex house; OFH is one family house; MFH is more family house; RH is raw house; V is villa; and CH is country house.

**Figure 19:**
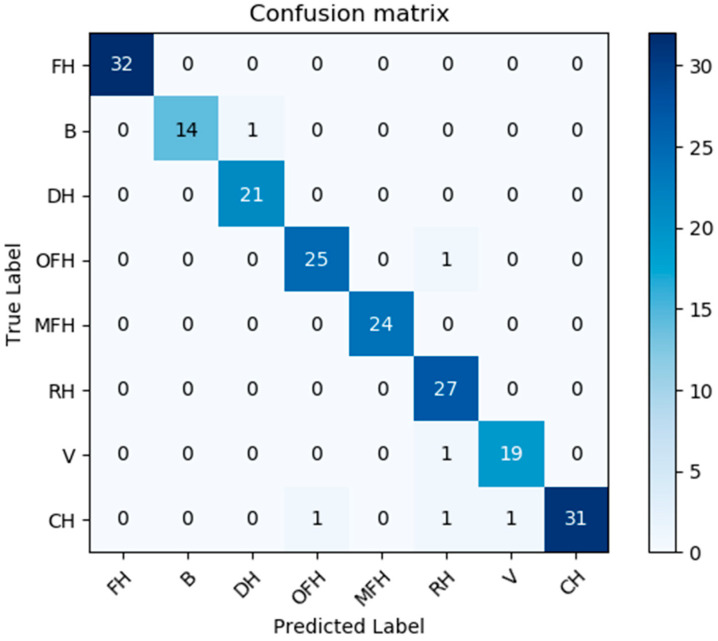
Top-2 Confusion matrix of the results obtained by Model III while using 200 test images. List of classes: FH is farmer house; B is bungalow house; DH is duplex house; OFH is one family house; MFH is more family house; RH is raw house; V is villa; and CH is country house.

Furthers, Table 2 presents the classification performance scores reached for the three models referred to in Table 1. Here we use the usual multi-dimensional classification performance metrics, namely accuracy, precision, F1-Score, and recall). Most of the classes have an interference/similarity problem with the class “country house”; and it is for this reason often mistaken with other house classes. Therefore, by changing our target function from “Top-1” to “Top-2”, our confusion matrix is changed/improved and most of the “similarity” problem is significantly solved/reduced (Figure 18 and Figure 19). Indeed, for practical use cases for which this classification may be relevant (e.g.: assessing the value of a given house for sales or for other purposes), using a “Top-2” classification may be fully sufficient.

In Table 3, the performance of our novel classifier model is compared to that of some very relevant previous/related works. These results clearly show/demonstrate that our novel method (which involves the above discussed multi-channel pre-processing features extraction) has the clearly best performance when compared to the various other models from the relevant recent literature.

One can see in Table 3 that our first CNN model without any additional preprocessing is much faster than all other models. However, after adding the pre-processing modules (for additional features) to our first model, the classification performance increases. This can also be seen in Table 1. In addition, both memory and processing time increase after adding the pre-processing layers.

In order to improve the overall classification performance of the housing prediction, the developed model has been divided into two modules: the pre-processors module, and the deep-learning module. The experimental results obtained show that this novel model significantly improves the classification performance. The price is, however, that more memory is consumed (although not very excessive) and the processing time slightly increases.

## 5. Conclusions

In this paper, a new CNN model for house types classification has been comprehensively developed and successfully validated. Its performance has also been compared to that of some recent very relevant previous works from literature. We can say clearly state that **our novel classification model has a much better performance w.r.t. classification performance** (i.e., accuracy, precision, recall, F1 score)**, memory usage, and even, to a large extent, also w.r.t. processing time.**

An objective justification/explanation of the superiority of our novel model presented in Figure 15 is also shown through the fact that adding more features through the different pre-processing units significantly increases the resulting related “NMI scores” metric. Indeed, we thus understand why adding additional features (through Sobel and Gabor filters) has resulted in significantly increasing the model’s classification performance (i.e., accuracy, precision, etc.)

Nevertheless, one could observe some misclassifications: a close analysis of the causes of them may inspire future works to reach a much better classification performance. Indeed, the fact of adding several pre-processing features extracting channels in the best-performing version of our novel model has some drawbacks: (a) it uses more memory compared to the (our first) model without those additional pre-processing channels; and (b) the training time is much longer, comparatively. 

In addition, a few classification errors have been observed. These misclassifications appear to be caused by the fact that certain house classes/types have a very strong similarity to one another. Examples: class “Villa” and class “Detached house”. This requires and inspires some future/further deep investigations and a subsequent better definition of house classes or, as a further option, a merging of some classes, which are visibly too similar to each other. All this does and shall have (in future works) the potential to make the overall resulting classification performance much more accurate and more robust against a series of imperfections of the input house images/photos. 

Also, the accuracy of the developed model can be further improved by extending by involving appropriately adapted inspirations involving, amongst others, a series of technical concepts and or paradigms such as the so-called “Adaptive Recognition” [41], “Dynamic Identification” [42], and “Manipulator controls” [43].

## Figures and Tables

**Table 1:** Normalized Mutual Information (NMI) Scores obtained for the input features for the various deep-learning models used (for the test data sets used in this work).

Model	CNN Model without Multi-Layer Channels (Figure 13)	CNN Model with Multi-Channel Features (Figure 14)	CNN Model with Multi-Channel Features (Figure 15)
NMI	79.5%	84.59%	88.19%

**Table 2:** Comparison of our novel model’s classification performance through different traditional metrics.

Model	CNN without Multi-Layer Channels (Figure 13)	CNN with Multi-Channel Features (Figure 15, Top-1)	CNN with Multi-Channel Features (Figure 15, Top-2)
Accuracy	86.5%	94.5%	96.4%
Precision	86.6%	94.2%	96.9%
F1 Score	86.7%	94.0%	96.1%
Recall	87.7%	93.9%	95.9%

**Table 3:** Comparison of our novel model’s performance with that of several other state-of-the-art classifier models published in previous/recent works from the relevant literature.

Model	Mathias (Involving SVM) [35]	Montoya Obesso (Involving CNN) [39]	ResNet-18 [40]	ResNet-34 [40]	CNN without Multi-Layer Channels (Figure 13)	CNN with Multi-Channel Features (Figure 15, Top-1)	CNN with Multi-Channel Features (Figure 15, Top-2)
Accuracy	77.1%	88.1%	78.1%	79.8%	86.5%	94.5%	96.4%
Precision	76.9%	87.7%	78.0%	80.1%	86.6%	94.2%	96.9%
F1-Score	76.5%	87.9%	77.8%	77.8%	86.7%	94.0%	96.1%
Recall	75.3%	88.2%	77.9%	75.8%	87.7%	93.9%	95.9%
Memory Usage	200 MB	100 MB	24 MB	34 MB	20 MB	67 MB	67 MB
Processing Time	100 ms	12 ms	11 ms	12 ms	9 ms	10 ms	10 ms
